# Correlation of Anthropometry With Plasma Atherogenicity Indices in Subjects With Type 2 Diabetes Mellitus

**DOI:** 10.7759/cureus.72414

**Published:** 2024-10-26

**Authors:** Jayant K Sahani, Ramesh Aggarwal, L. H Ghotekar, Anupam Prakash, Kuldeep Singh, Prashasti Gupta

**Affiliations:** 1 Internal Medicine, Lady Hardinge Medical College, New Delhi, IND

**Keywords:** anthropometric measures, atherogenic co-efficient, atherogenic index, body mass index: bmi, cardiovascular risk (cvr), glycated hemoglobin (hba1c), lipid profiles, type2 diabetes mellitus, urinary-albumin-creatinine-ratio (uacr), waist circumfernce

## Abstract

Introduction

Diabetes mellitus is characterized by chronic hyperglycemia due to insulin deficiency, leading to complications in vital organs. Among these, dyslipidemia is common, presenting as low high-density lipoprotein cholesterol (HDL-c), high triglycerides (TG), Apolipoprotein-B (Apo-B), and small dense low-density lipoprotein (sdLDL) predominance, collectively known as diabetic dyslipidemia. To assess the atherogenic risk in individuals with type 2 diabetes, the atherogenic index of plasma (AIP) and atherogenic coefficient (AC) provide valuable insights beyond routine lipid tests. AIP, calculated as log (serum TG/serum HDL-c), correlates positively with the occurrence and severity of diabetic microvascular complications. The AC ((total cholesterol (TC)-HDL-c)/HDL-c) serves as an atherogenicity marker. Waist circumference (WC), reflecting central adiposity and body mass index (BMI), are directly related to both AIP and AC, making them useful non-invasive tools to monitor atherogenicity and predict cardiovascular disease (CVD) risk independently of each other in subjects with type 2 diabetes mellitus.

Material and methods

This was an observational cross-sectional study conducted in the Department of Medicine of a tertiary care hospital. It included 100 type 2 diabetes mellitus patients more than 18 years of age, including both males and females.

Observation and results

In our study, there were 42 (42%) males and 58 (58%) females. The mean WC of males and females were 105.40 and 100.98 cm, respectively. The mean for BMI, glycated hemoglobin (HbA1c), and urine albumin-to-creatinine ratio (UACR) was 28.83 kg/m^2^, 8.58%, and 100.62 mg/g, respectively. There was positive Pearson's correlation between AIP and WC of males and females (r = 0.324 and 0.269), AC and WC of males and females (r = 0.139 and 0.097), BMI and AIP (r = 0.350), BMI and AC (r = 0.214), HbA1c and AIP (r = 0.207), HbA1c and AC (r = 0.216), UACR and AIP (r = 0.218), and UACR and AC (r = 0.237).

Conclusion

This study concludes that there is a positive correlation between anthropometric measures, such as WC and BMI, and plasma atherogenicity indexes, including the AIP and AC. This finding suggests that clinicians can effectively use these non-invasive measurements (BMI and WC) to estimate the presence of dyslipidemia and atherogenicity in patients with type 2 diabetes mellitus during routine outpatient care. Early identification of these risk factors allows for timely lifestyle interventions such as dietary modifications and increased physical activity, which could potentially reduce the risk of future cardiovascular diseases.

## Introduction

Lipid abnormalities in patients with diabetes, often termed “diabetic dyslipidemia,” are typically characterized by high total cholesterol (TC), high triglycerides (TG), low high-density lipoprotein cholesterol (HDL-c) and increased levels of small dense low-density lipoprotein (sdLDL) particles [1,2]. The sdLDL particles have a greater tendency than normal low-density lipoprotein (LDL) to bind with glycoproteins, allowing them to more easily deposit on arterial walls. This leads to the buildup of cholesterol and hinders the clearance of lipoprotein particles from the blood, increasing the risk of arteriosclerosis and cardiovascular disease. Measuring LDL particle size is typically complex and expensive, involving techniques like polyacrylamide gradient gel electrophoresis and gel filtration chromatography, which are not widely available in general hospitals. In 2000, Dobiasova and Frohlich proposed a simpler parameter called the AIP, which is a logarithmic transformation of the ratio of TG concentration to HDL cholesterol. AIP is inversely correlated with LDL particle size. Therefore, AIP, which quantifies the relationship between TG and HDL cholesterol and has a negative correlation with LDL particle size, could be a useful surrogate for small LDL particle size in assessing diabetic dyslipidemia and the risk of type 2 diabetes [3]. AIP is positively associated with both the occurrence and severity of diabetic microvascular complications [4].

The atherogenic coefficient (AC) is a ratio calculated as (TC-HDL-c)/HDL-c. The AC compares the cholesterol content of atherogenic and protective lipoproteins, providing a broader atherogenicity assessment than LDL-C alone. Hence, the AC is considered a marker of atherogenicity [5].

Evident correlations were observed between anthropometric and clinical variables and lipid profiles. Waist-to-height ratio (WHtR) and waist circumference (WC) could be used as a simple and non-invasive method for the detection of dyslipidemia as an important risk factor for cardiovascular complications [6].

## Materials and methods

Study design

An observational, cross-sectional study was conducted in a tertiary care hospital in India.

Study subjects and sampling

A sample of 100 adult (age ≥ 18 years, both males and females) patients was taken over a 13-month period from November 2022 to February 2024. The study was approved by the Institutional Ethics Committee.

Data collection

Informed written consent was obtained from each patient. Patients with type 2 diabetes mellitus presenting to the inpatient and outpatient medicine departments were included in the study. Non-ambulatory, pregnant, critically ill, on anti-lipidemic drugs, known cases of any chronic illnesses or hyperlipidemia, and those patients in whom anthropometry cannot be measured correctly were excluded. The patient’s demographic details, along with health-related information, with particular emphasis on details about diabetic status, WC, and body mass index (BMI), were recorded in a pre-designed structured proforma. A fasting venous blood sample of approximately 5 mL was extracted and used for the analysis of fasting blood glucose (FBG), glycated hemoglobin (HbA1c), and serum lipid profiles comprising total serum cholesterol (TC), LDL-c, TG, and HDL-c. The lipid profile was measured by colorimetry (AU5800, Beckman Coulter, Brea, CA) and expressed as mg/dL. HbA1c was measured using the ion-exchange high-performance liquid chromatography (HPLC) method. UACR (Urine Albumin-to-Creatinine Ratio) was measured using colorimetry by an autoanalyzer (AU5800) and expressed as mg/gm.

Definition of terms

An abnormal lipid profile was defined as having a TC ≥ 200 mg/dL, HDL-c < 40 mg/dL, LDL-c ≥ 130 mg/dL, and TG ≥ 150 mg/dL [7]. Patients were classified as diabetic if their HbA1c >6.5% (48 mmol/mol) or if they had a previous clinical diagnosis of diabetes [8]. Patients were considered overweight if their BMI was between 25.0 and 29.9 kg/m2 [9,10]. They were categorized as obese if their BMI was greater than or equal to 30.0 kg/m2 [10]. A WC of up to 94 cm for males and up to 80 cm for females was considered normal [10].

Statistical analysis

Data were coded and entered into Microsoft Excel (Microsoft® Corp., Redmond, WA), then exported to the Statistical Package for Social Science (SPSS) ( IBM SPSS Statistics for Windows, IBM Corp., Version 25, Armonk, NY) for further analysis. The normality of the data was tested, and the data were reported as mean and standard deviation (SD) for continuous variables and as percentages for categorical variables. Variables were compared using an independent sample t-test after confirming a normal distribution. The strength of the association between pairs of variables was assessed using Pearson’s correlation coefficient. A correlation was considered statistically significant if the p-value was less than 0.05. Finally, the results were summarized and presented in the form of text, tables, and frequencies.

## Results

Demographic characteristics of subjects

**Figure 1:**
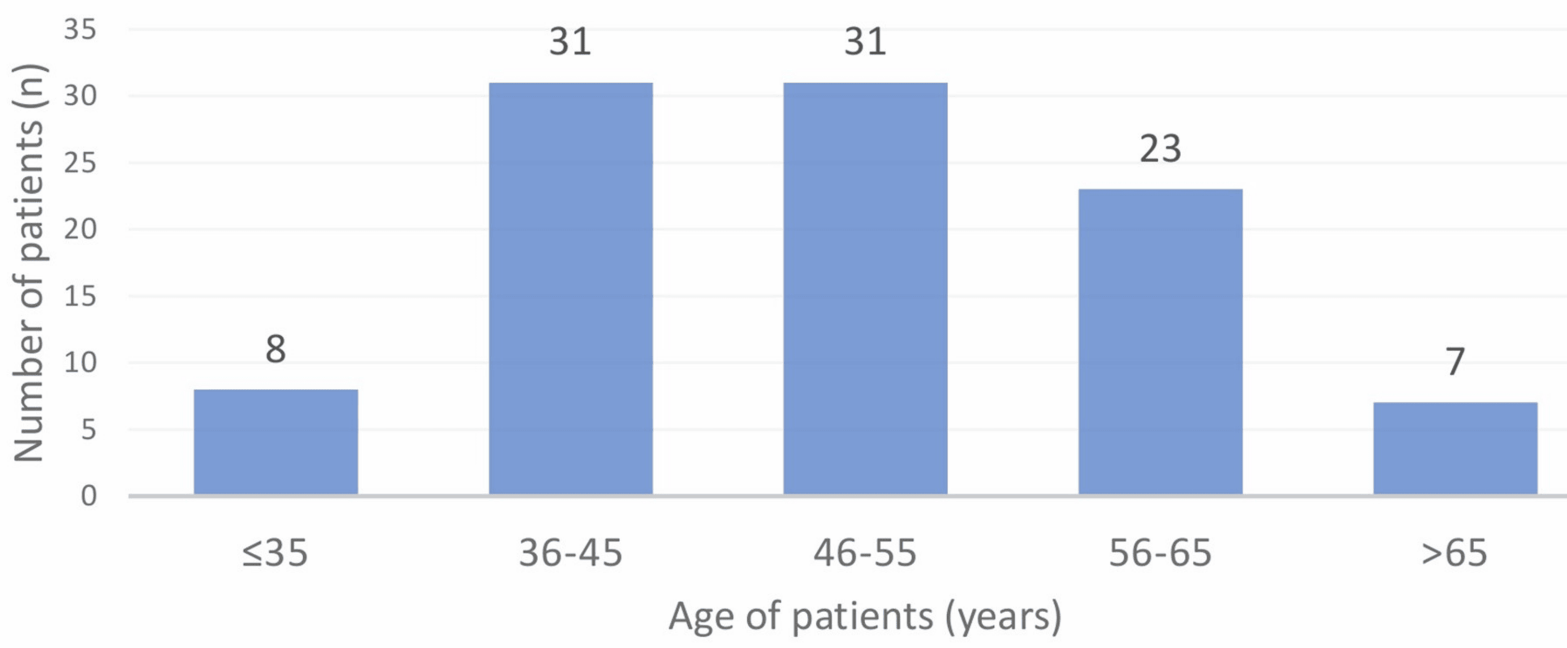
Distribution of cases according to age of the patient

**Figure 2:**
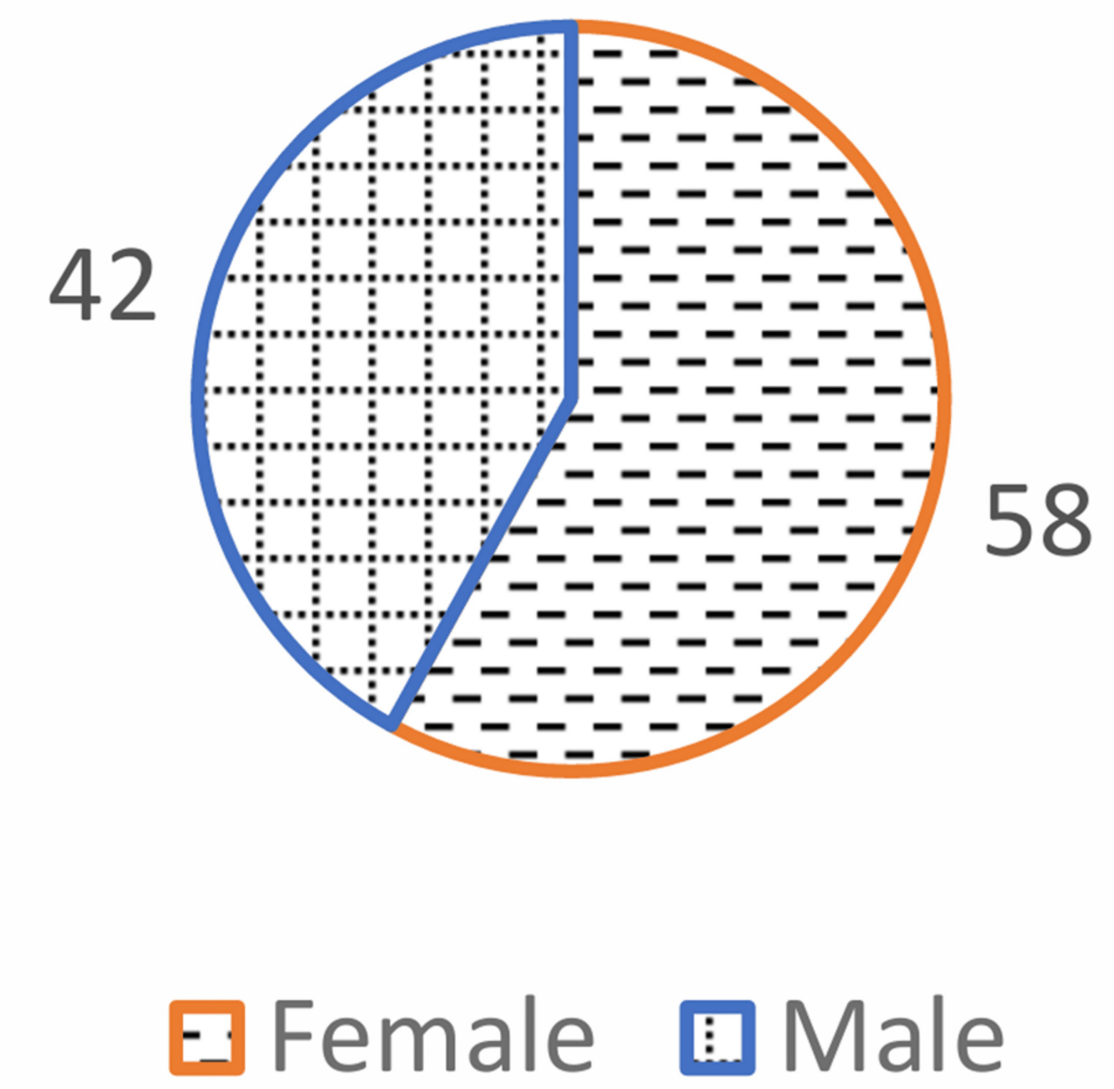
Distribution of cases according to sex of the patient

The study population consisted of 100 type 2 diabetes mellitus subjects. The average age of the study group was 49.71 ± 10.62 years, ranging from 21 to 76 years. Out of 100 study patients, 58 (58%) were female, and 42 (42%) were male (Figure 1 and Figure 2).

Mean values

The mean WC of male and female subjects was 105.40 ± 9.27 cm and 100.98 ± 10.27 cm, respectively. The majority of subjects, i.e., 42 (42%), fell under the overweight-obese II category (BMI ≥ 30 kg/m^2^), followed by 38 (38%) subjects in the overweight-obese I category (BMI = 25-29.99 kg/m^2^). There were 11 (11%) subjects with a normal BMI (18.5-22.99 kg/m^2^). Out of 100 subjects, the majority, i.e., 55 (55%), had HbA1c values greater than or equal to 8%, followed by 34 (34%) subjects with HbA1c values ranging from 6.5% to 8%. Seventy (70%) subjects had deranged serum cholesterol, 66 (66%) had deranged serum TG, 54 (54%) had deranged serum LDL, and 61 (61%) had deranged serum HDL cholesterol levels. Seventy-seven (77%) had microalbuminuria with a mean value of 100.62 ± 58.9 mg/g.

Correlations between WC and plasma atherogenicity

**Figure 3:**
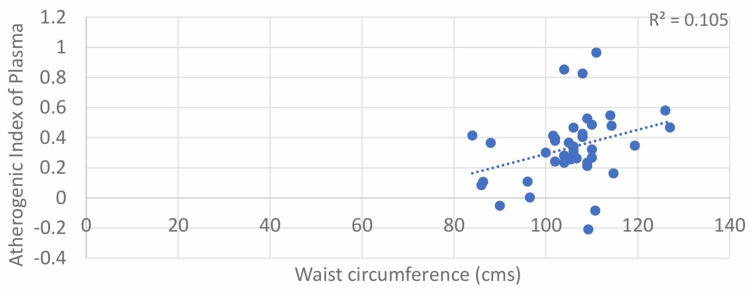
Correlation between waist circumference (male) and atherogenic index of plasma (AIP)

**Figure 4:**
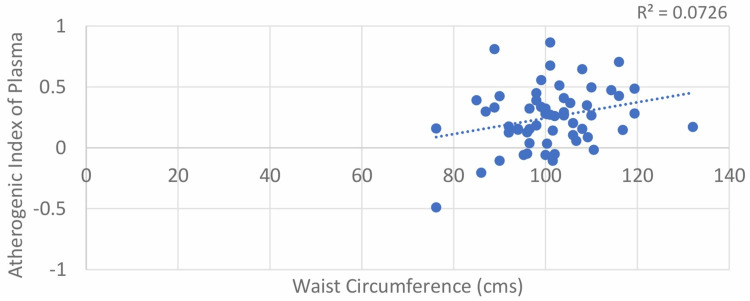
Correlation between waist circumference (female) and atherogenic index of plasma (AIP)

The results of our study show a moderately positive correlation between WC and AIP in both males and females, with Pearson’s correlation coefficient values of 0.324 and 0.269, respectively. The correlation was statistically significant, with p-values of 0.036 and 0.041 for both males and females, respectively (Table 1; Figure 3 and Figure 4).

**Table 1:** Correlation of waist circumference with plasma atherogenicity

Waist circumference (cm)	Correlation (r) with atherogenic index of plasma	p-value	Correlation (r) with atherogenic coefficient	p-value
Male	0.324	0.036	0.139	0.380
Female	0.269	0.041	0.097	0.468

**Figure 5:**
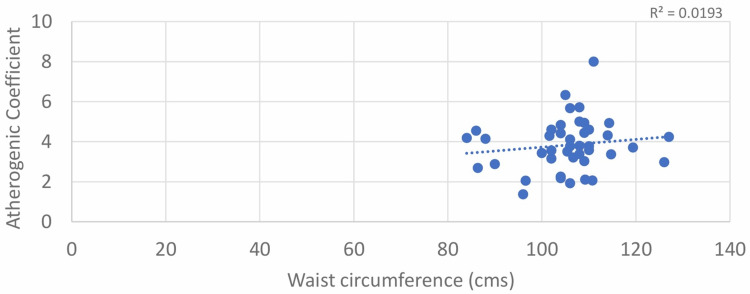
Correlation between waist circumference (male) and atherogenic coefficient (AC)

**Figure 6:**
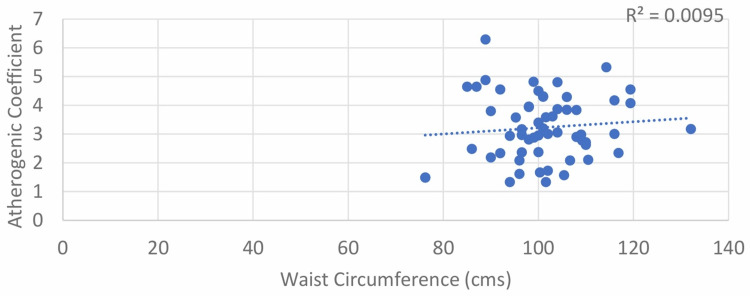
Correlation between waist circumference (female) and atherogenic coefficient (AC)

A weak positive correlation was found between WC and AC for both males and females, with Pearson’s correlation coefficient values of 0.139 and 0.097, respectively; however, the results were not statistically significant with p > 0.05 (Table 1; Figure 5 and Figure 6). 

Correlations between BMI and plasma atherogenicity

**Figure 7:**
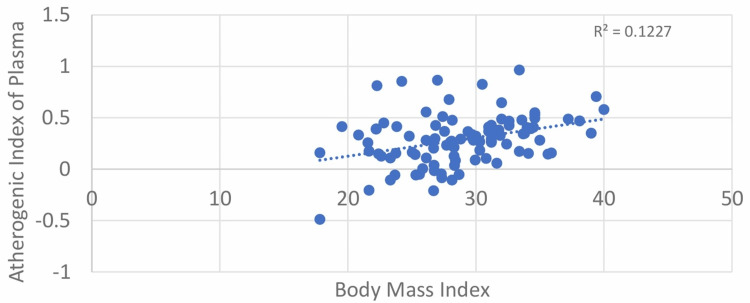
Correlation between BMI and atherogenic index of plasma (AIP)

**Figure 8:**
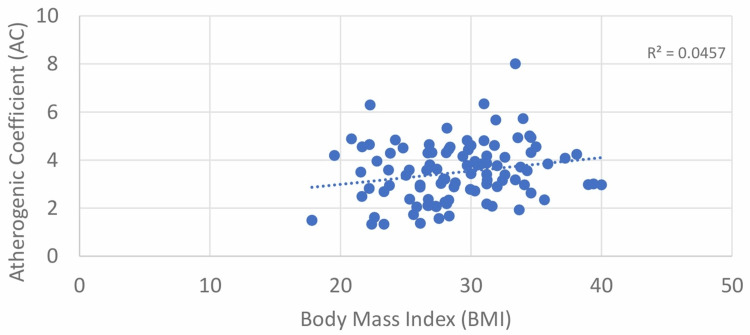
Correlation between BMI and atherogenic coefficient (AC)

In our study, a strong positive correlation between BMI and AIP was found with Pearson’s correlation coefficient value of 0.350. The correlation was statistically significant, with a p-value of <0.001 (Table 2, Figure 7). For the correlation between BMI and AC, the results obtained had a Pearson’s correlation coefficient value of 0.214, which was statistically significant with a p-value of 0.033 (Table 2, Figure 8).

**Table 2:** Correlation of BMI with plasma atherogenicity

BMI	Atherogenic index of plasma	Atherogenic coefficient
Pearson’s correlation coefficient	0.350	0.214
p-value	<0.001	0.033

Correlations between glycemic status and plasma atherogenicity

**Figure 9:**
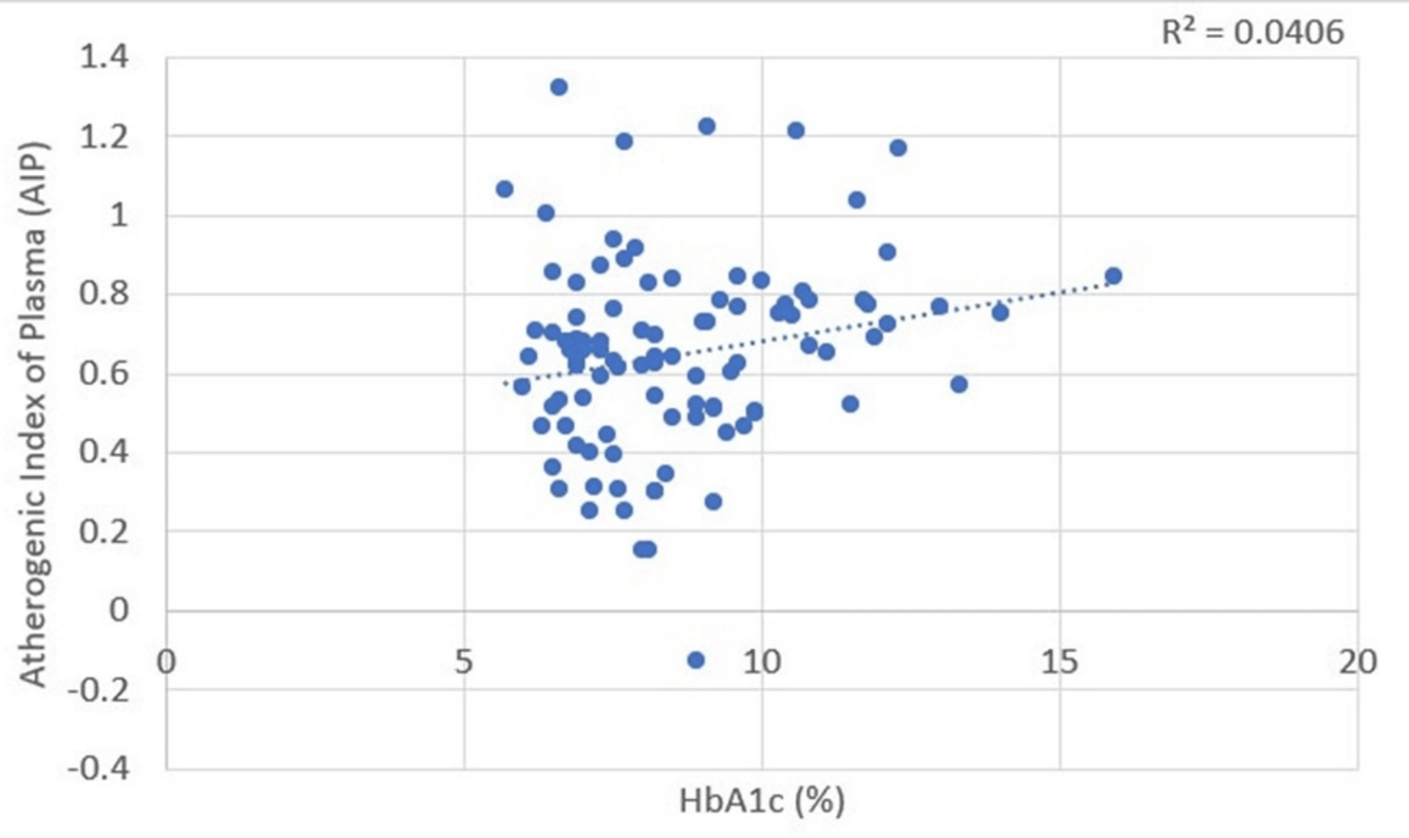
Correlation between glycemic status (glycated hemoglobin (HbA1c)) and atherogenic index of plasma (AIP)

**Figure 10:**
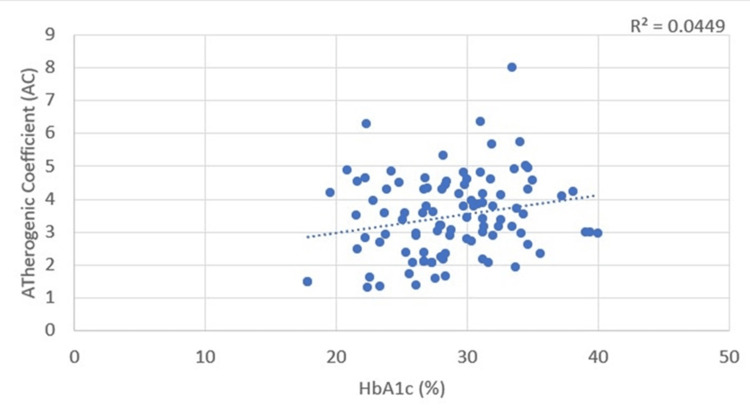
Correlation between glycemic status (glycated hemoglobin (HbA1c)) and atherogenic coefficient (AC)

A positive correlation was found between HbA1c and the AIP, with a Pearson’s correlation coefficient value of 0.207. This correlation was statistically significant, with a p-value of 0.039 (Table 3, Figure 9). HbA1c correlated significantly (p = 0.031) with the AC, with a Pearson’s correlation coefficient value of 0.216 (Table 3, Figure 10).

**Table 3:** Correlation of glycemic status (glycated hemoglobin (HbA1c)) with plasma atherogenicity

HbA1c	Atherogenic index of plasma	Atherogenic coefficient
Pearson’s correlation coefficient	0.207	0.216
p-value	0.039	0.031

Correlations between UACR and plasma atherogenicity

**Figure 11:**
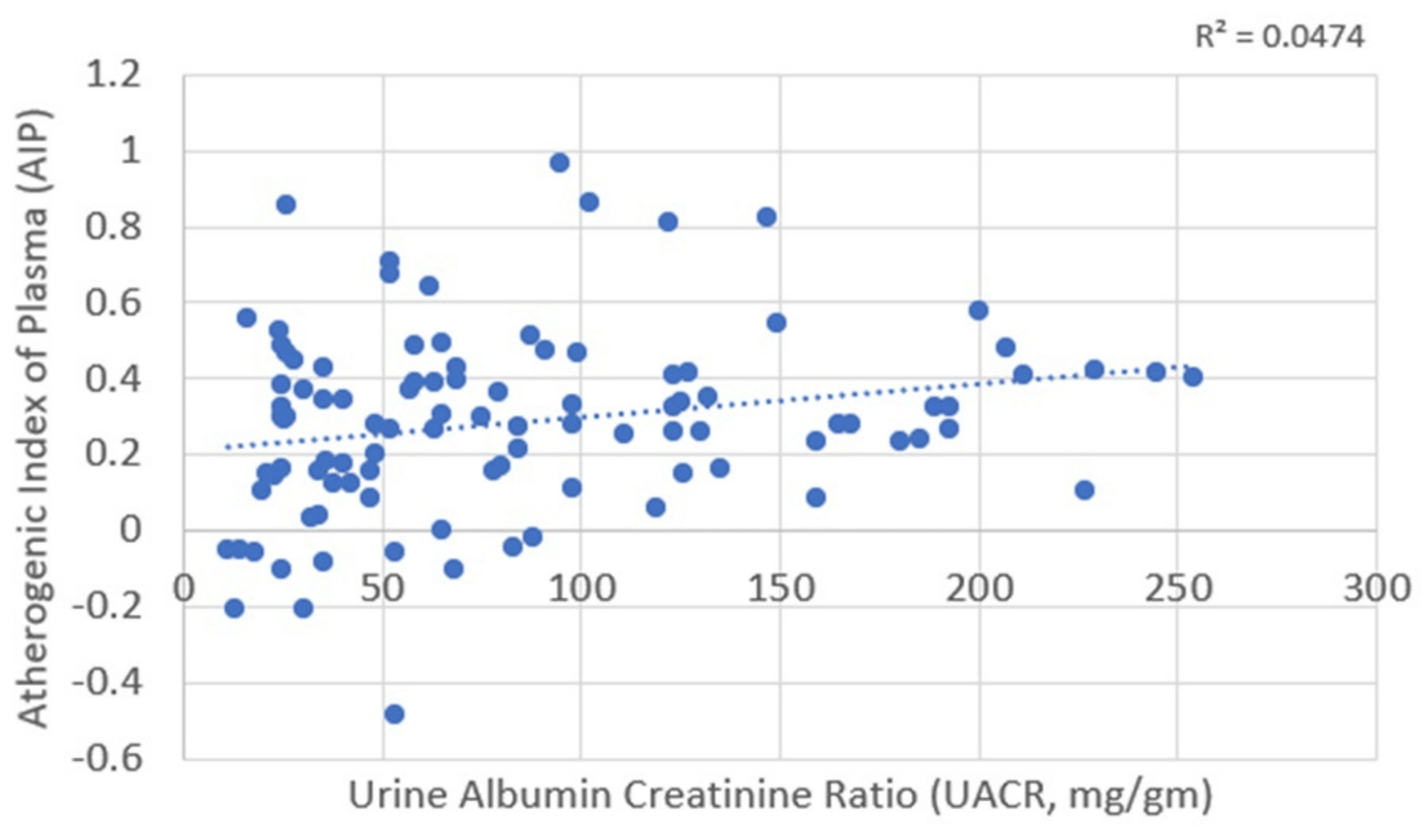
Correlation between urine albumin-to-creatinine ratio (UACR) and atherogenic index of plasma (AIP)

**Figure 12:**
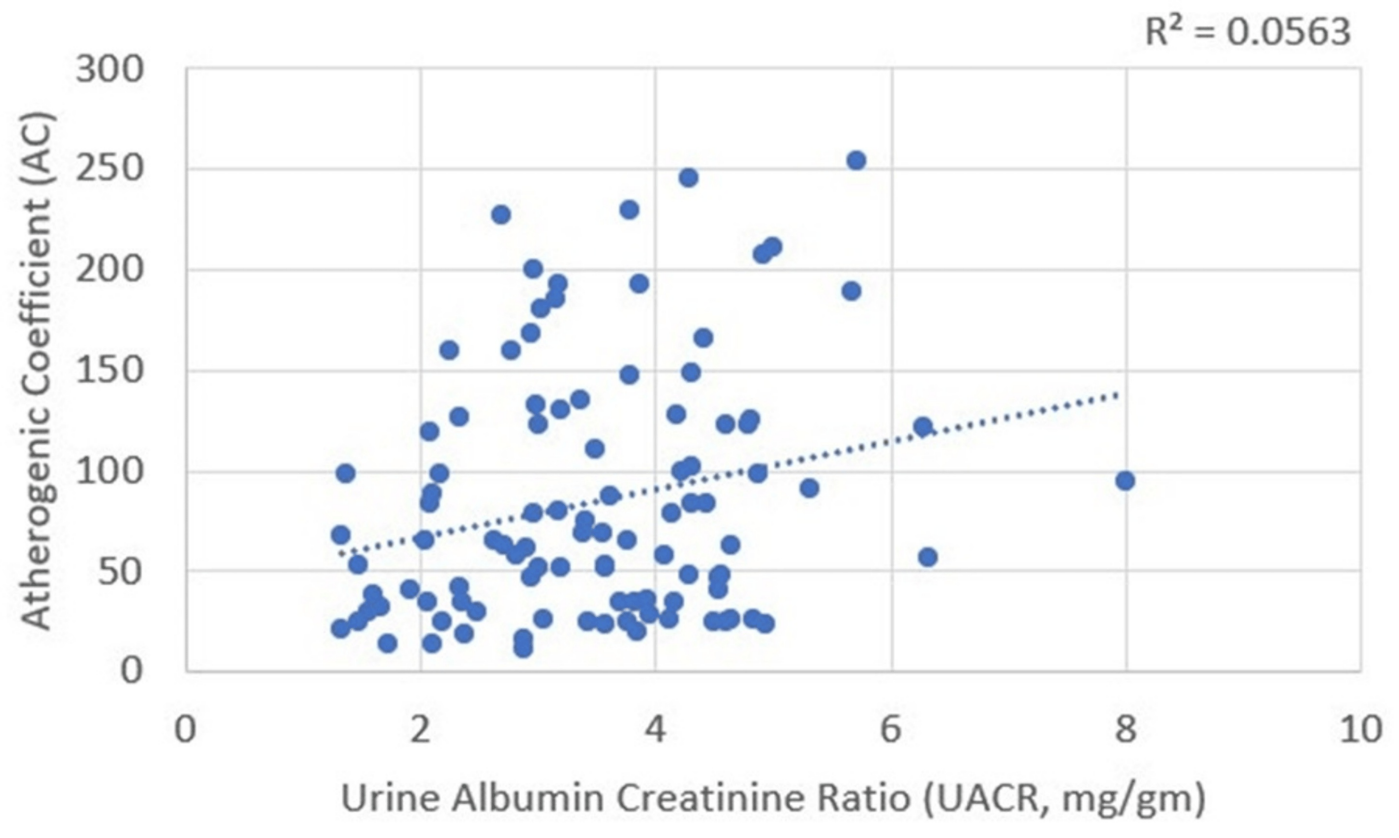
Correlation between urine albumin-to-creatinine ratio (UACR) and atherogenic coefficient (AC)

UACR correlates positively with the AIP and AC, with Pearson’s correlation coefficient values of 0.218 and 0.237, respectively. These correlations were statistically significant, with p-values of 0.030 and 0.017 for AIP and AC, respectively (Table 4; Figure 11 and Figure 12).

**Table 4:** Correlation of UACR with plasma atherogenicity UACR, urine albumin-to-creatinine ratio

UACR	Atherogenic index of plasma	Atherogenic coefficient
Pearson’s correlation coefficient	0.218	0.237
p-value	0.030	0.017

## Discussion

Type 2 diabetes begins with a gradual decline in insulin effectiveness, causing the body to struggle with glucose transport into tissues and leading to insulin receptor desensitization. To compensate, the body produces more insulin, resulting in reduced insulin sensitivity in target tissues. This insulin resistance contributes to dyslipidemia, high blood pressure, and altered glucose metabolism. Insulin resistance disrupts hormones that control hunger and satiety, leading to increased calorie intake, weight gain, and a higher BMI. Excess calories are stored as abdominal fat, increasing WC. Obesity leads to insulin resistance and worsens diabetes and dyslipidemia due to adipokines and chronic inflammation from adipose tissue [11].

WC and plasma atherogenicity (AIP and AC)

Shen et al. found a linear correlation between WC and AIP, with a 1 cm increase in WC leading to a 0.0175 rise in AIP (r = 0.371). Anandkumar et al. reported a similar correlation (r = 0.369) in 60 female patients [12]. Niroumand et al. observed a correlation of 0.35 (p < 0.001) with no gender differences [13]. Zhen Li et al. found a lower correlation in type 2 diabetes (r = 0.129, p < 0.001) [14]. Our study also found positive correlations between WC and AIP in both males (r = 0.324) and females (r = 0.269).

The correlation between WC and AC, although non-significant, was positive, as found in the study conducted by Aryl et al. [15]. In our study also, the correlation between WC and AC was positive but non-significant in both males and females (r = 0.139; p = 0.380 and 0.097; p = 0.468, respectively).

BMI and plasma atherogenicity (AIP and AC)

Our study found a significant positive correlation between AIP and BMI (r = 0.350, p < 0.001), consistent with findings by Li et al. (r = 0.182, p < 0.001) and Bo et al. (r = 0.25) [14].

According to Mallika et al., there was a significant and positive correlation between BMI and AC [16]. Additionally, in our investigation, there was a significant and positive correlation (r = 0.214; p < 0.05) between BMI and AC.

HbA1c and plasma atherogenicity (AIP and AC)

Anjankar et al. showed a positive correlation between AIP and HbA1c in patients with good (r = 0.490, p = 0.005) and poor glycemic control (r = 0.568, p = 0.0001) [17]. Our study also found a positive and significant correlation between both AIP (r = 0.207, p = 0.039) and AC (r = 0.216, p = 0.031) with HbA1c.

In type 2 diabetes, insulin resistance leads to increased TG and free fatty acids and decreased HDL-c, resulting in dyslipidemia. Dyslipidemia, in turn, can trigger insulin resistance and poor glycemic control through inflammation and stress. This creates a vicious cycle where each condition worsens the other [18].

This suggests that HbA1c, in addition to monitoring glycemic control, can also provide insights into lipid levels and atherogenic risk.

UACR and plasma atherogenicity (AIP and AC)

Several studies highlight the relationship between AIP and microalbuminuria in patients with type 2 diabetes. Xu et al. found higher AIP levels in patients with nephropathy and retinopathy, identifying AIP as a predictor for microalbuminuria and diabetic nephropathy [4]. Qi et al. reported that AIP has a good predictive value for microalbuminuria (AUC = 0.772) [19]. Atalay et al. found a statistically significant correlation between AIP and microalbuminuria (r = 0.221, p < 0.01) [20]. Additionally, our study identified significant positive correlations between AIP (r = 0.218, p = 0.030) and AC (r = 0.237, p = 0.017) with the UACR.

Hyperlipidemia results from abnormal lipid intake and/or metabolism, leading to increased levels of cholesterol, TG, or both (combined hyperlipidemia). When the body's energy intake consistently exceeds its ability to store fat in adipose tissue, excess lipids start to accumulate in non-adipose tissues like muscles, liver, kidneys, and pancreas. This condition is termed “ectopic lipid accumulation.” In the kidneys, these lipids can deposit in various cell types, including mesangial cells, podocytes, and proximal tubule epithelial cells [21].

Limitations

The cross-sectional design of this study restricts the evaluation of causality, and the single-center population may have an impact on generalizability. Additional long-term research is required to validate these results and investigate the causal connections between atherogenic indices and anthropometric measurements. Further insights into the clinical utility of BMI and WC reduction interventions in managing cardiovascular risk in patients with type 2 diabetes may come from research on their effects on AIP and AC.

## Conclusions

This study concludes that there is a positive correlation between anthropometric measures such as WC and BMI and plasma atherogenicity indexes, including the AIP and AC. This finding suggests that clinicians can effectively use these non-invasive measurements (BMI and WC) to estimate the presence of dyslipidemia and atherogenicity in patients with type 2 diabetes mellitus during routine outpatient care. Early identification of these risk factors allows for timely lifestyle interventions such as dietary modifications and increased physical activity, which could potentially reduce the risk of future cardiovascular diseases.
